# Supplementary material for: Modulating the Surface Properties of Lithium Niobate Nanoparticles by Multifunctional Coatings Using Water-in-Oil Microemulsions
Source: Nanomaterials (Basel). 2023 Jan 28;13(3):522. doi: 10.3390/nano13030522 (PMC9921616; doi:10.3390/nano13030522)
Supplement: Supplementary file 1 [file nanomaterials-13-00522-s001.zip › nanomaterials-2148259-supplementary.pdf]

Supporting information

Modulating the Surface Properties of Lithium Niobate Nanoparticles by Multifunctional Coatings Using Water-in-Oil Microemulsions

Adrian Gheata[1] and Alessandra Spada[1], Manon Wittwer[1, 2], Ameni Dhouib[3], Emilie Molina[3], Yannick Mugnier[3], Sandrine Gerber-Lemaire[1,*]

[1]Institute of Chemical Sciences and Engineering, Ecole Polytechnique Fédérale de Lausanne, Group for Functionalized Biomaterials, EPFL SB ISIC SCI-SB-SG, Station 6, CH-1015 Lausanne, Switzerland
[2]Département de chimie, École normale supérieure, PSL University, 75005 Paris, France
[3]SYMME, Université Savoie Mont-Blanc, Annecy F-74000, France

*E-mail: sandrine.gerber@epfl.ch

Table of contents

Details on experimental protocols and characterization instrumentation S-3
Synthesis of 4-oxo-4-((3-(triethoxysilyl)propyl)amino)butanoic acid (APTES-COOH) S-4
Synthesis and characterization of **Alys** S-6
Analytical data for **Talys**: ^{1}H and ^{13}C, HRMS and FTIR S-8
Microemulsion DLS data S-10
Characterization of bare LNO NPs S-11

Details on experimental protocols and characterization instrumentation

Reagent-grade solvents (Fluka, Riedel-de-Haën) and chemicals (Aldrich, Acros, Fluka, Sigma, Maybridge, TCI Chemicals, Apollo, abcr and Fluorochem) were used without further purification. All reactions were performed in flame-dried glassware under an inert atmosphere of nitrogen. All products were dried under vacuum (10^{-2} bar) before analytical characterization. Reactions were monitored by thin layer chromatography (TLC) on pre-coated aluminum plates SiO_2 60 F254 (Merck, Darmstadt, Germany). The compounds were visualized by 254 nm light or stained with solutions of $KMnO_4$ or ninhydrin. Purifications were performed by flash chromatography (FC) on silica gel (Merck N° 9385 silica gel 60, 230-400 mesh, particle size 40-63 μm). NMR spectra were recorded on Bruker Avance III-400, Bruker Avance-400 or Bruker DRX-400 spectrometers (Bruker, Billerica, MA, USA) at rt. ^{1}H frequency is at 400.13 MHz, ^{13}C frequency is at 100.62 MHz. Chemical shifts are reported downfield from tetramethylsilane. ^{1}H signals are reported in ppm with the internal chloroform signal at 7.26 ppm, the internal methanol signal at 3.31 ppm or the internal DMSO signal at 2.50 ppm as references. ^{13}C-NMR signals are reported in ppm with the internal chloroform signal at 77.00 ppm, the internal methanol signal at 49.00 ppm or the internal DMSO signal at 39.5 as internal references. The resonance multiplicity is described as s (singlet), d (doublet), t (triplet), q (quartet), quin (quintet), m (multiplet). Broad signals are indicated as br. Coupling constants (J) are given in hertz (Hz). Ultrasonication was performed on Elmasonic P 120 H or Branson 1800 Ultrasonic Cleaner sonicators. IR spectra were recorded on a Nicolet 6700 FT-IR Spectrophotometer from Thermo Fisher Scientific with KBr pellets. The spectra are reported in cm^{-1}. The qualitative accurate masses were measured by ESI-TOF using the Xevo G2-S QTOF (Waters) and nanoESI-FT-MS using the Elite™ Hybrid Ion Trap-Orbitrap (ThermoFisher) Mass Spectrometer. Measurements of hydrodynamic size and zeta potential were performed with Malvern NanoZ Instrument. Absorbance and fluorescence measurements were performed with BioTek Synergy H1 multi-mode reader instrument between 200 and 700 nm with increment of 1 nm in a 96-well plate (Corning® UV-Transparent microplate). Protein coating procedures were carried out using the MSC-100 Thermo Shaker Incubator (LabGene) at 4°C, at stirring speeds of 750 rpm. Centrifugation cycles were performed using HERAEUS Biofuge 13 centrifuge and Beckman Coulter Allegra X-30 centrifuge. Ultrafiltration was performed using Amicon® Ultra15-10K Centrifugal Filter Devices (Millipore®). Scanning transmission electron microscopy (STEM) and energy-dispersive X-ray spectroscopy (EDX) were performed at the Interdisciplinary Centre for Electron Microscopy (CIME, EPFL, Lausanne, Switzerland) on a FEI Tecnai Osiris (200 kV).

Preparation of 4-oxo-4-((3-(triethoxysilyl)propyl)amino)butanoic acid (APTES-COOH)

Protocol adapted from the synthetic procedure reported in [79]:

Vuilleumier, J. Functionalization of Second Harmonic Generation Nanoparticles for Theranostic Applications, EPFL, Lausanne, **2019**. https://doi.org/10.5075/epfl-thesis-7330

Scheme S1. *Synthetic route towards* ***APTES-COOH***

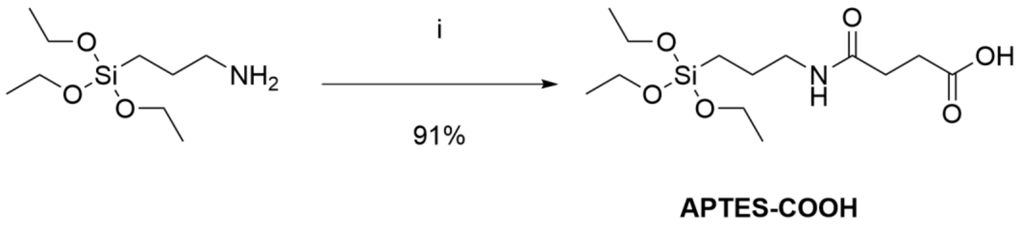

Reagents and conditions: i) Succinic anhydride, dioxane, r.t., 1h.

Succinic anhydride (0.58 g, 5.89 mmol, 1.25 eq) was dissolved in dioxane (5 mL) under stirring at 60°C. The solution was cooled down to r.t. and APTES (1.1 mL, 4.7 mmol, 1.0 eq) was added dropwise. The reaction mixture was stirred at r.t. for 1 h. The solution was filtered and the solvent was removed under reduced pressure, to afford **APTES-COOH** (0.99 g, 3.1 mmol, 68% yield) as a yellow oil. The analytical data were in accordance with previously reported data.

^{1}H NMR (400 MHz, Chloroform-*d*): δ 6.24 (s, 1H), 3.87 – 3.73 (m, 6H), 3.21 (dq, *J* = 11.9, 6.5 Hz, 2H), 2.64 (ddd, *J* = 7.5, 5.7, 4.2 Hz, 2H), 2.47 (ddd, *J* = 8.4, 5.7, 2.4 Hz, 2H), 1.60 (ddt, *J* = 13.8, 11.6, 4.7 Hz, 2H), 1.31 – 1.07 (m, 9H), 0.65 – 0.54 (m, 2H).

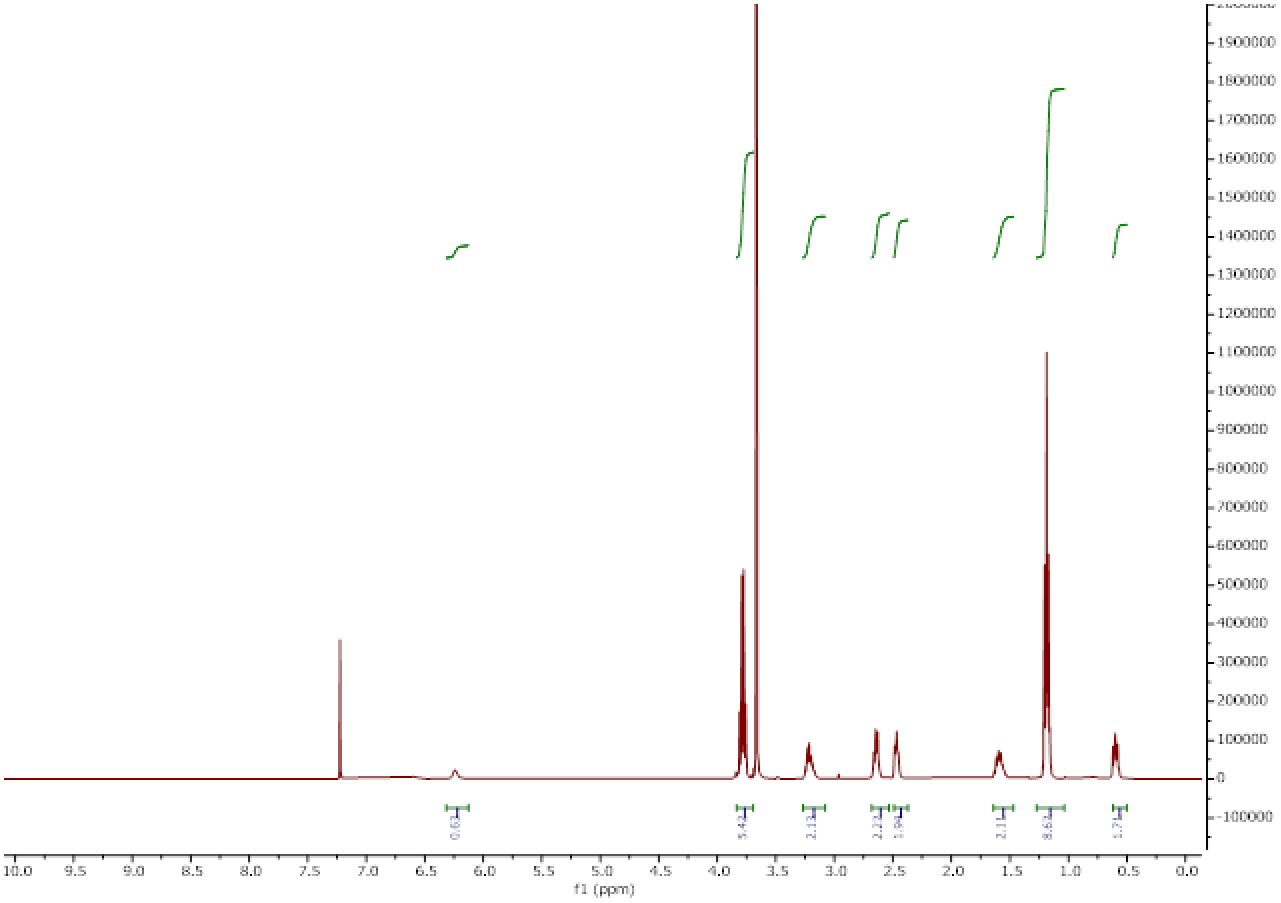

^{13}**C NMR** (100 MHz, Chloroform-*d*): δ 172.5, 67.0, 58.6, 58.4, 42.1, 30.8, 22.6, 18.4, 18.3, 18.2, 7.7.

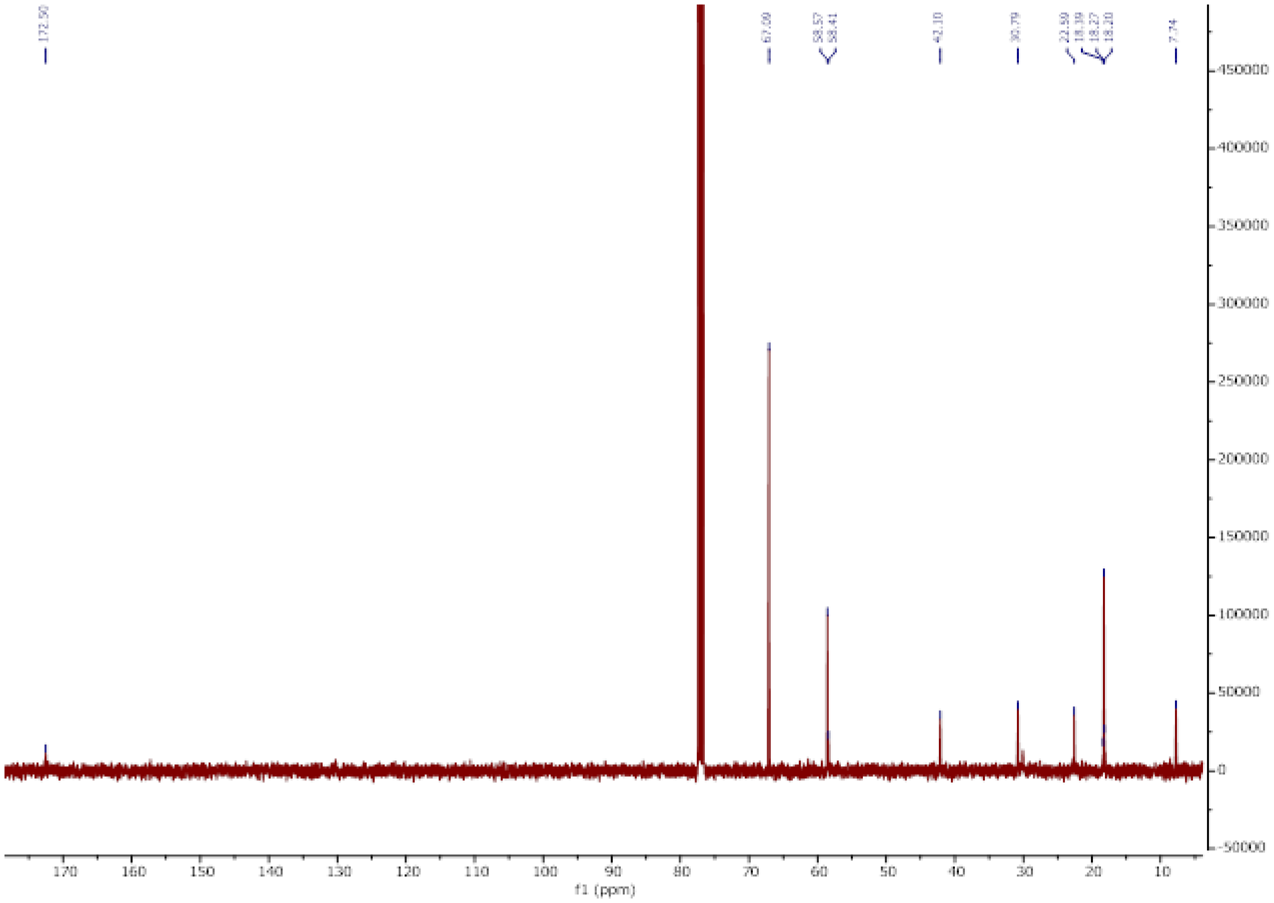

HRMS (ESI/QTOF) m/z: $[M - H]^-$ Calcd for $C_{13}H_{26}NO_6Si^-$ 320.1535; Found 320.1540.

FTIR (neat, cm^{-1}): 3263, 3091, 2973, 2928, 2884, 1784, 1726, 1669, 1645, 1564, 1433, 1394, 1364, 1346, 1297, 1255, 1225, 1190, 1167, 1101, 1076, 1005, 952, 848, 775, 679.

Preparation of azidolysine (Alys) amino acid

Scheme S2. *Synthetic route toward Fmoc-Alys-OH*

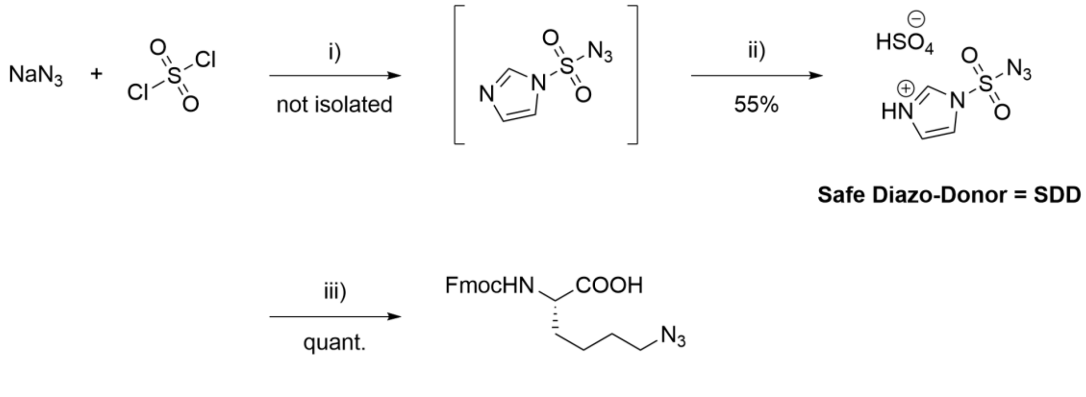

Fmoc-Alys-OH

Reagents and conditions: i) imidazole, dry EtOAc, 0 °C, 20 h; ii) H_2SO_4, dry EtOAc, 0 °C, 1 h; iii) Fmoc-Lys-OH, $CuSO_4$, $NaHCO_3$, MeOH / H2O 4:1, r.t, 5 h.

Imidazole-1-sulfonyl azide, hydrogen sulfate salt: SDD

Adapted from protocols described in [80]:

Fischer, N., Goddard-Borger, E.D., Greiner, R., Klapo¨tke, T.M., Skelton, B.W. and Stierstorfer, J. Sensitivities of some imidazole-1-sulfonyl azide salts. *J. Org. Chem.* **2012**, *77,* 1760-1764. https://doi.org/10.1021/jo202264r

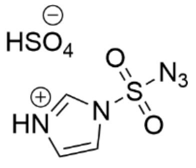

NaN_3 (5.0 g, 77 mmol, 1.0 eq) was suspended in dry EtOAc (80 mL) and cooled to 0 °C under argon. Sulfuryl chloride (6.2 mL, 77 mmol, 1.0 eq) was added dropwise, the reaction was stirred and allowed to warm to r.t for 18 h. The mixture was cooled again to 0 °C and imidazole (10.0 g, 146 mmol, 1.9 eq) was slowly added while maintaining an inert atmosphere. The reaction was stirred at 0 °C for 3 h, and then basified by very slow addition of a saturated aqueous solution of $NaHCO_3$ (100 mL). The aqueous phase was discarded and the organic phase was washed with H_2O (80 mL). After separation, the organic phase was dried over $MgSO_4$ and filtered. The filtrate was put again under argon and cooled to 0 °C. Concentrated H_2SO_4 (4.1 mL, 77 mmol, 1.0 eq) was added dropwise, and the mixture was stirred for 1 h at 0 °C. The reaction was left to slowly warm to r.t and filtrated, the solid was washed with cooled EtOAc. After drying under high vacuum for 24 h, the product was afforded as a white solid (11.5 g, 42 mmol, 55%), and was stored under argon at -20 °C. The analytical data were in accordance with previously reported data.

^{1}H NMR (400 MHz, DMSO-d_6): δ = 14.30 (s, br, NH^+), 12.42 (s, br, $HSO4^-$), 9.06 (t, CH, J = 1.1 Hz), 8.69 (dd, CH, J_1 = 0.8 Hz, J_2 = 1.5 Hz), 7.98 (dd, CH, $J_{1/2}$ = 1.5 Hz), 7.66 (d, CH, J = 1.2 Hz), 7.37 (dd, CH, J_1 = 0.9 Hz, J_2 = 1.7 Hz).

HRMS (nanochip-ESI/LTQ-Orbitrap) m/z: $[M + Na]^+$ Calcd for $C_3H_4N_5O_2SNa^+$ 174.0079; Found 174.0080.

Fmoc-Alys-OH

Adapted from protocols described in [81]:

Sminia, T.J. and Pedersen, D.S. Azide-and alkyne-functionalised α-and β3-amino acids. *Synlett* **2012**, *23*, 2643-2646. https://doi.org/ 10.1055/s-0032-1317445

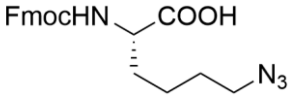

Fmoc-Lys-OH.HCl (1.0 g, 2.7 mmol, 1.0 eq), $CuSO_4$ (70 mg, 0.41 mmol, 0.15 eq) and $NaHCO_3$ (0.8 g, 9.5 mmol, 3.5 eq) were suspended in MeOH / H2O 4:1 (10 mL). The SDD (0.9 g, 3.3 mmol, 1.2 eq) was added and the pH was maintained to 8-9 by addition of NaOH 1M in H_2O (continuous monitoring with pH meter). The reaction mixture was stirred for 5 h at r.t. The crude was concentrated under reduced pressure to remove MeOH, then diluted with sulfate buffer pH 2 (50 mL) and extracted with EtOAc (50 mL, 3 times). The combined organic phases were washed with brine (50 mL, two times), dried over $MgSO_4$, filtered and concentrated under reduced pressure to afford the product as a yellow oil (1.1 g, 2.7 mmol, quant). The analytical data were in accordance with previously reported data.

^{1}H NMR (400 MHz, Chloroform-*d*): δ = 7.77 (2H, d, *J* = 8 Hz, Ar), 7.60 (2H, d, *J* = 8 Hz, Ar), 7.42 (2H, t, *J* = 7.5 Hz, Ar), 7.32 (2H, t, *J* = 7.5 Hz, Ar), 5.27 (1H, br d, *J* = 8 Hz, NH), 4.60-4.22 (3H, m, $FmocCH_2$ and Į-CH), 4.24 (1H, t, *J* = 7 Hz, Fmoc-CH), 3.31 (2H, t, *J* = 6.5 Hz, CH_2N_3), 2.01-1.43 (6H, m, 3 × CH_2).

HRMS (nanochip-ESI/LTQ-Orbitrap) m/z: $[M + Na]^+$ Calcd for $C_{21}H_{22}N_4O_4Na^+$ 417.1554; Found 417.1533.

Analytical data for Talys: ^{1}H and ^{13}C, HRMS and FTIR

^{1}H NMR (400 MHz, DMSO-d_6): δ = 8.95 (s, 1H), 8.49 (d, J = 8.1 Hz, 1H), 8.25 – 8.18 (m, 2H), 7.88 (d, J = 8.2 Hz, 1H), 4.40 (td, J = 8.2, 5.1 Hz, 1H), 4.28 (dtd, J = 20.1, 8.3, 5.2 Hz, 2H), 3.81 – 3.67 (m, 2H), 3.56 (d, J = 1.7 Hz, 2H), 3.36 – 3.31 (m, 2H), 3.31 – 3.25 (m, 6H), 1.71 – 1.62 (m, 3H), 1.52 (dq, J = 9.9, 6.7, 6.2 Hz, 9H), 1.33 (p, J = 8.1 Hz, 6H).

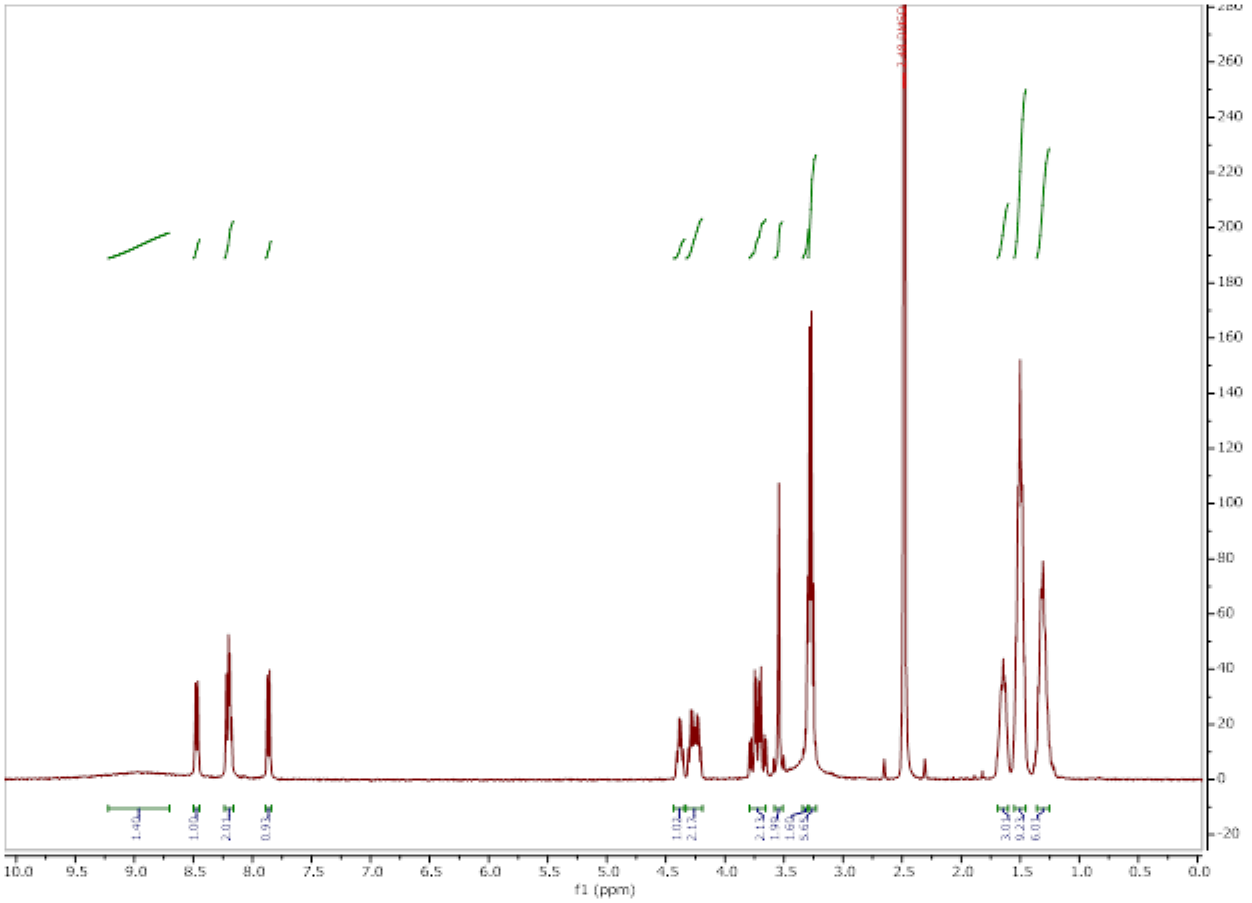

^{13}C NMR (100 MHz, DMSO-d_6): δ = 172.08, 171.61, 171.54, 171.51, 166.39, 52.94, 52.67, 52.28, 51.02, 51.00, 50.96, 41.14, 32.50, 32.24, 31.81, 28.37, 28.34, 28.26, 23.07, 22.79, 22.65.

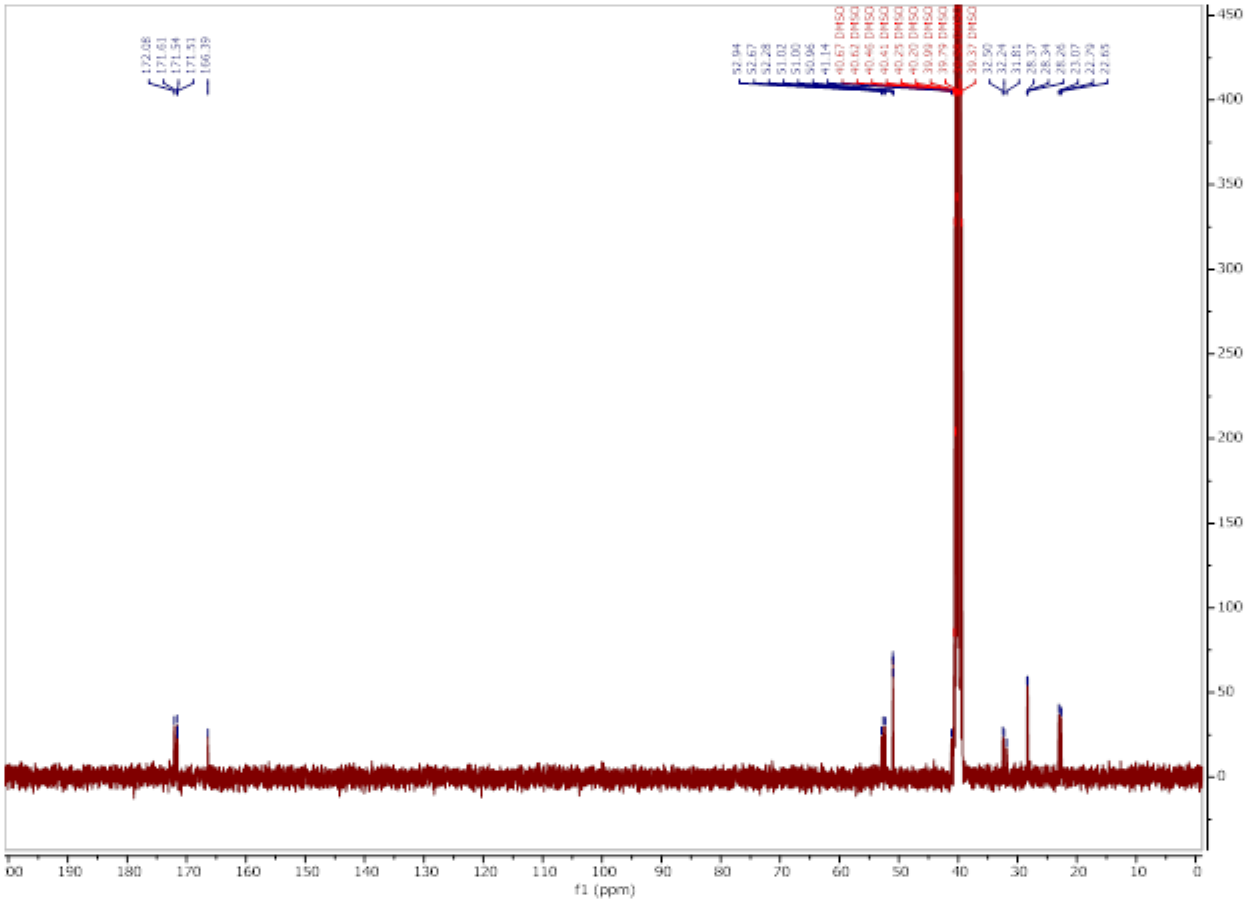

HRMS (nanochip-ESI/LTQ-Orbitrap) m/z: $[M + H]^+$ Calcd for $C_{22}H_{39}N_{14}O_6N^+$ 595.3172; Found 595.3181.

FTIR (neat, cm^{-1}): 3275, 2942, 2093, 1696, 1662, 1626, 1530, 1429, 1251, 1199, 1182, 1136, 923, 837, 799, 722.

Microemulsion DLS data

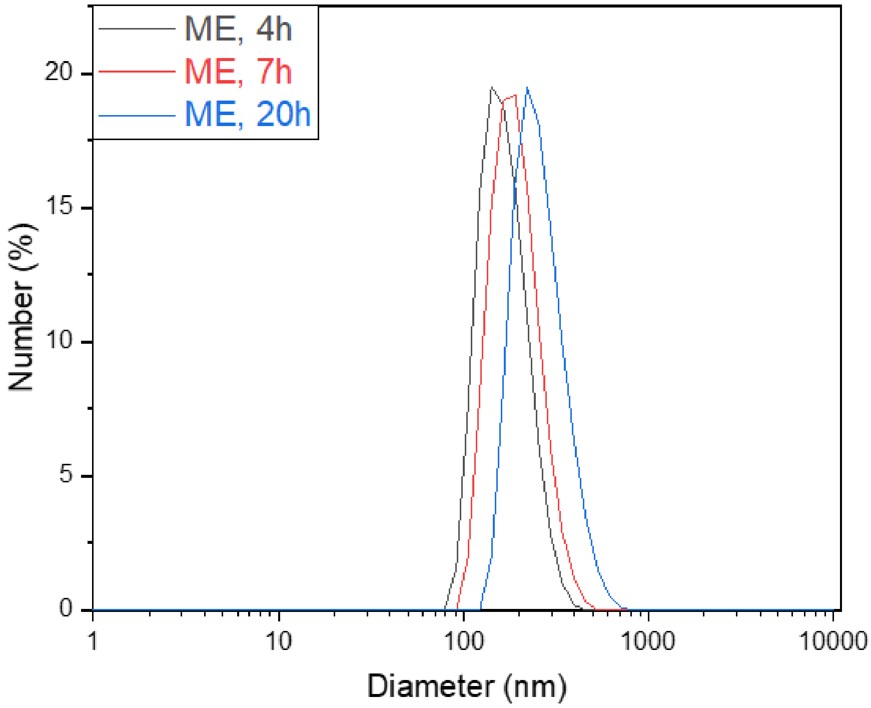

Figure S1: DLS characterization of the ME size evolution in time

Characterization of bare LNO NPs

Solvothermal preparation of lithium niobate (LNO) nanocrystals in a 23 mL Teflon-lined stainless-steel autoclave (Parr Instruments Co., Moline, IL, USA) was done at 235 °C for 1 day. In a typical synthesis, niobium ethoxide (154 uL, 0.6 mmol) was first diluted under Argon in 3.75 mL of absolute ethanol before addition of lithium ethoxide (600 uL, 0.6 mmol) yielding to the bimetallic lithium niobium ethoxide $LiNb(OCH_2CH_3)_6$ at 0.133M in ethanol. Homogenization with magnetic stirring for about 5 minutes was then carried out outside of the glove box resulting in a transparent yellow solution. Addition under stirring of 2.25 mL of 1,4-Butanediol results in ligand exchange with the ethoxy groups. To promote condensation at room temperature, a mixture of 100 uL of distilled water and 900 uL of absolute ethanol was then added dropwise thus keeping a homogeneous solution without precipitation. The reaction medium was then kept under vigorous stirring for 24 hours before the solvothermal treatment. After cooling down to room temperature, the monolith typically obtained was isolated from the reaction medium upon centrifugation (13,500 rpm) before being re-dispersed twice in ethanol for additional washing and centrifugation.
X-ray diffraction (XRD) characterizations were obtained in the 5-120° 2θ range with the Co $K\alpha_1$ and $K\alpha_2$ radiations of a PANalytical X'Pert3 powder diffractometer (Malvern Panalytical, Palaiseau, France) equipped with a rotating zero-background silicon sample holder. The apparent nanocrystal size S_{hlk} derived from the peak broadening along each (hkl) direction was calculated after extraction of the integrated intensities within FullProf according to the Le Bail global fitting procedure. A pseudo-Voigt function and a platelet morphology were assumed to fit the eventual anisotropic broadening of the diffraction peaks with consideration of the Lorentzian and Gaussian parts in the FWHM and after careful assessment of the instrument resolution from a Silicon calibration powder. Assessment of the mean nanocrystal size and shape polydispersity from TEM observations and data treatment of the XRD profiles is detailed below. The Le Bail profile refinement of the powder XRD pattern leading to a residual χ^2 value at 1.86 is depicted in Figure S2 from which the derived crystallite sizes along each [hkl] direction is at about 35 nm.

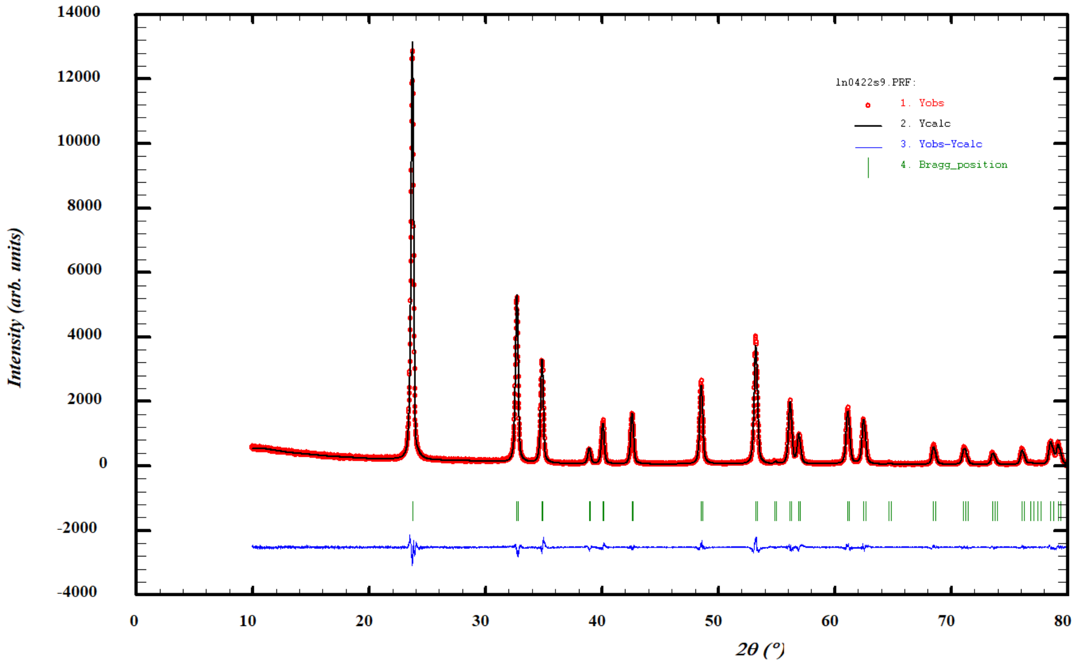

Figure S2: Le Bail profile refinement of the powder XRD pattern of LNO nanocrystals obtained with 2.25 mL of 1,4-Butanediol as co-solvent.

The mean XRD size obtained from the refinement is to be considered though with caution because of the size and shape polydispersity revealed from the TEM observations of bare LNO nanocrystals (Figure S3). After counting 100 individual nanoparticles, the corresponding size distribution histogram is centered at 53 nm with a 17 nm standard deviation. The larger particle size at about 100 nm and the DLS size distribution in number (centered at about 100 nm as well) of bare LNO nanocrystals was thus considered to calculate the number of ammonia droplets.

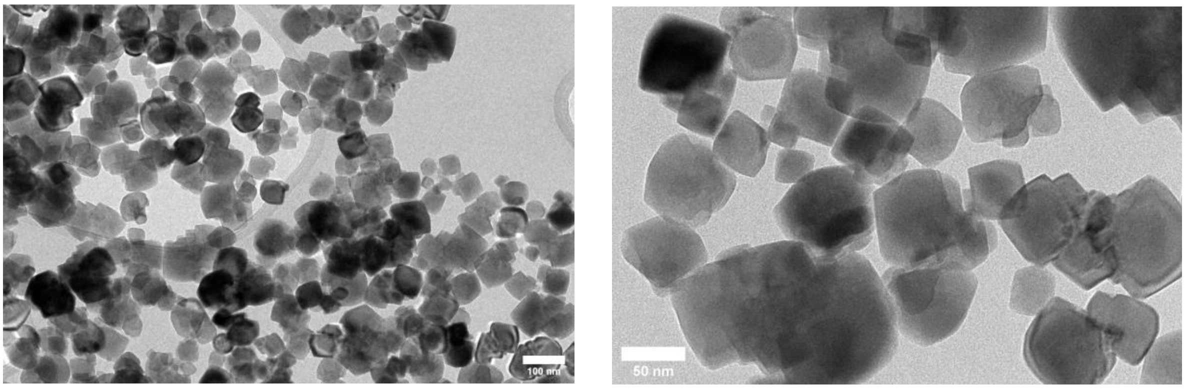

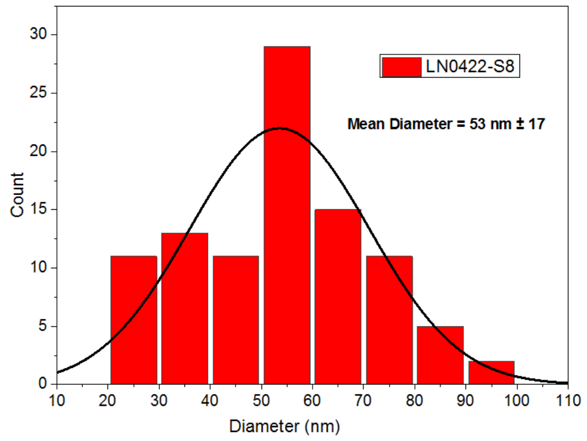

Figure S3: Representative TEM images and associated size distribution histogram of LNO nanocrystals obtained with 2.25 mL of 1,4-butanediol as co-solvent.
